# Ice nucleation triggered by negative pressure

**DOI:** 10.1038/s41598-017-16787-3

**Published:** 2017-11-30

**Authors:** Claudia Marcolli

**Affiliations:** Institute for Atmospheric and Climate Science, ETH Zurich, Zurich, Switzerland

## Abstract

Homogeneous ice nucleation needs supercooling of more than 35 K to become effective. When pressure is applied to water, the melting and the freezing points both decrease. Conversely, melting and freezing temperatures increase under negative pressure, i.e. when water is stretched. This study presents an extrapolation of homogeneous ice nucleation temperatures from positive to negative pressures as a basis for further exploration of ice nucleation under negative pressure. It predicts that increasing negative pressure at temperatures below about 262 K eventually results in homogeneous ice nucleation while at warmer temperature homogeneous cavitation, i. e. bubble nucleation, dominates. Negative pressure occurs locally and briefly when water is stretched due to mechanical shock, sonic waves, or fragmentation. The occurrence of such transient negative pressure should suffice to trigger homogeneous ice nucleation at large supercooling in the absence of ice-nucleating surfaces. In addition, negative pressure can act together with ice-inducing surfaces to enhance their intrinsic ice nucleation efficiency. Dynamic ice nucleation can be used to improve properties and uniformity of frozen products by applying ultrasonic fields and might also be relevant for the freezing of large drops in rainclouds.

## Introduction

Crystal nucleation has remained inaccessible to direct observation due to its stochastic nature which renders the exact time and location of occurrence unpredictable. However, the industrial interest in a better control of crystallization processes to improve product quality and uniformity is high^1^. One of the most important and best investigated crystallization process is freezing. Apart from homogeneous ice nucleation occurring at a random location in the volume of liquid water, ice nucleates heterogeneously on ice-inducing surfaces^2^.

Micrometer-sized water drops can be cooled to about 235 K before homogeneous ice nucleation sets in. When 2 mm^3^ drops of pure distilled water are cooled, freezing usually occurs above 250 K due to heterogeneous nucleation on surfaces that facilitate the orientation of water molecules into an ice-like structure. Extreme precautions need to be taken to avoid the presence of ice-nucleating impurities in order to achieve large supercooling. To reach freezing temperatures of around 240 K for water volumes of 3–50 mm^3^ cooled in glass capillaries, Mossop^3^ needed to exclude room air during distillation, capillary preparation and filling. Conversely, freezing seems to be facilitated when supercooled water is agitated. Observations are frequent that bottled water cooled below the melting temperature freezes when the bottle is shaken, hit on a table or opened up. While it is clear that the bottle wall and particles in the water are able to induce ice nucleation, it is puzzling why some kind of additional agitation is needed to initiate freezing.

Supercooling below 235 K is reached when aqueous solutions instead of pure water are cooled, because the melting and the freezing temperatures of water are both depressed, as a consequence of the reduced water activity in the presence of solutes. Koop *et al*.^4^ derived curves of constant homogeneous ice nucleation rates as a function of water activity by shifting the melting curve by a constant amount to higher water activity. In the absence of specific interactions between the solute and the ice-nucleating surface, a constant offset in water activity is also able to describe the heterogeneous ice nucleation with increasing solution concentration^5^. Increased pressure has a similar effect on melting and freezing as reduced water activity. In contrast to gases, liquids can be put under tension by stretching them. Stretched water withstands negative pressure due to the cohesive forces between the molecules in the liquid. Water under tension is metastable with respect to the two-phase state of water in coexistence with its vapor. Here we explore whether the occurrence of negative pressure may account for freezing when water is agitated.

## Results and Discussion

### The influence of pressure on ice and bubble nucleation

**Figure 1:**
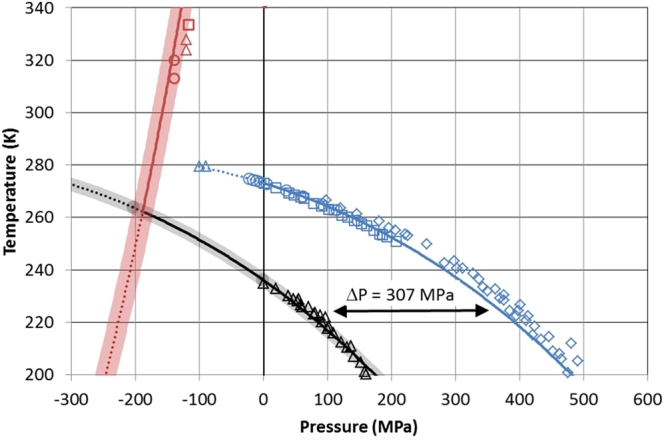
Pressure dependence of melting (blue) and freezing (black) temperatures of ice I and cavitation (red) temperatures. Note that on this scale, ambient pressure (0.1 MPa) coincides with the zero pressure line. Melting point measurements of ice I are from Kanno *et al*.^26^ (blue squares), Mishima^27^ (blue diamonds), Henderson and Speedy^51^ (blue circles) and Roedder^6^ (blue triangles). Freezing temperatures of ice I (black triangles) are from Kanno *et al*.^26^. For simplicity, melting and freezing data of other ice polymorphs are not shown in this graph. Cavitation temperatures are from Zheng *et al*.^10^ (red circles), Azouzi *et al*.^12^ (red triangles) and Shmulovich *et al*.^11^ (red square). The blue line is a fit of the measured melting temperatures (blue symbols) as a function of pressure (P) using the following equation: T (K) = 557.2 − 273*exp((300 + P(MPa))^2^/2270000). The dotted portion is the extrapolation to negative pressure. The black curve represents a homogeneous ice nucleation rate of 10^8^ cm^−3^s^−1^ obtained by shifting the blue curve by ΔP = 307 MPa to lower values. Note, that this curve is not a fit to the measured freezing temperatures (black triangles). The dotted portion indicates the range where the homogeneous cavitation rate exceeds the homogeneous ice nucleation rate. The grey shaded area reflects the uncertainty of the nucleation rate in pure water. The red curve gives the cavitation temperature according to CNT for a homogeneous bubble nucleation rate of 10^8^ cm^−3^s^−1^. The dotted portion indicates the range where homogeneous ice nucleation rates exceed homogeneous cavitation rates. The red shaded area represents estimated uncertainties in this value.

Figure 1 shows that pressure applied to water decreases the melting (blue) and the freezing temperatures (black). The blue curve is a fit to the experimentally determined melting temperatures as a function of pressure. The black curve is derived by shifting the blue melting curve by *ΔP* = 307 MPa to lower pressure and represents ice nucleation with a rate coefficient of about 10^8^ cm^−3^s^−1^ required for freezing of micrometer-sized water volumes within about a second (see theory section for more details). The good agreement of this line with the measured freezing temperatures shows that the effect of pressure on freezing can be described by shifting the melting curve by a constant offset in pressure, analogous to the constant offset that relates melting and freezing temperatures as a function of water activity^4^. When the melting and freezing curves are extrapolated to negative pressure, higher melting and homogeneous freezing temperatures are predicted than under normal pressure. The increased chemical potential of water under tension is manifested by an elevation of the melting point of ice. Roedder^6^ measured a melting point of almost 280 K of ice enclosed in micrometer-sized pockets inside quartz crystals when the vapor phase was eliminated during freezing (blue triangles in Fig. 1). He estimated that the liquid water phase in equilibrium with the ice phase needs to be at a negative pressure possibly exceeding 100 MPa for such a melting point elevation. However, large tensions can only be obtained in the absence of cavitation. Cavitation pressure as a function of temperature can be calculated based on classical nucleation theory (CNT, see theory section for a description of bubble nucleation by CNT) and is given as the red curve in Fig. 1 for a nucleation rate coefficient of 10^8^ cm^−3^s^−1^. The cavitation curve intersects the ice nucleation curve at T = 262 ± 4 K and P = −189 ± 15 MPa. CNT predicts that at temperatures below this intersection ice nucleation occurs at a higher rate than cavitation and should therefore be the dominating process when water is put under tension. However, ice nucleation and cavitation rates have to be considered as uncertain by at least two orders of magnitude as indicated by the grey and red shaded areas, respectively.

### Measurements of negative pressure

Negative pressure develops under various circumstances. Some of them have been used to determine the limiting negative pressure that can be reached before cavitation occurs. One way to generate tension is centrifugation of a liquid in a tube. The centrifugal force during rotation at high speed eventually disrupts the liquid column. Briggs^7^ applied high speed rotation to a z-shaped glass capillary which was open at both ends. With this method, he reached a minimum pressure of −27.7 MPa at the center of rotation at 280 K.

Acoustic waves successively pressurize and stretch liquids. When large enough amplitudes are applied, cavitation occurs. Galloway^8^ investigated cavitation induced by radially symmetric standing waves of 20–40 kHz in a spherical resonator at room temperature. Without degassing, he reached negative pressures close to −1 MPa before an air bubble appeared. Cleaned and degassed water could withstand negative pressures down to −20 MPa for up to a minute before a vapor bubble nucleated. Galloway also noted, that 100 times greater pressures could be imposed for some seconds without causing cavitation. High transient tensions can be reached locally by tightly focused travelling waves. When clean, degassed water was exposed to such waves, cavitation pressure was found to be a monotonically increasing function of temperature from −34 MPa at 273 K to −5 MPa at 473 K^9^.

The Berthelot method invented by Marcellin Berthelot more than 150 years ago measures the maximum negative pressure a liquid sealed in a tube can reach before the tension is released by cavitation. The experiment starts at ambient temperature with a sealed tube that is almost completely filled with a liquid. On heating, the liquid expands until it completely fills the tube. During cooling, the adhesion to the tube walls prevents the liquid from contracting leading to increasing tension which is eventually relieved by cavitation. To deduce the maximum negative pressure from the volume change when cavitation occurred, the equation of state that relates pressure with density needs to be known. The highest tensions in water can be reached by applying the Berthelot method to microscopic volumes. Zheng *et al*.^10^ produced water inclusions in quartz crystals by heating quench fractured quartz crystals in water to high temperatures and pressures to trap water in small pockets when the fractures healed. Upon cooling to 313–320 K, the water in such microscopic inclusions could be stretched to around −140 MPa before a bubble formed (red circles in Fig. 1), consistent with the prediction by CNT and therefore likely to represent the homogeneous cavitation pressure. Although these values have been confirmed by other researchers using the same method (red square^11^ and red triangles^12^ in Fig. 1), they have been questioned^9^ because such negative cavitation pressures are far from being reached by the other techniques summarized above and because the equation of state has to be extrapolated to negative pressure to evaluate the experiments. Nevertheless, it is likely that experiments performed in larger volumes suffer from ubiquitous impurities that induce heterogeneous cavitation before homogeneous cavitation becomes effective just as in the case of ice nucleation^3^.

### Ice nucleation triggered by negative pressure

**Figure 2:**
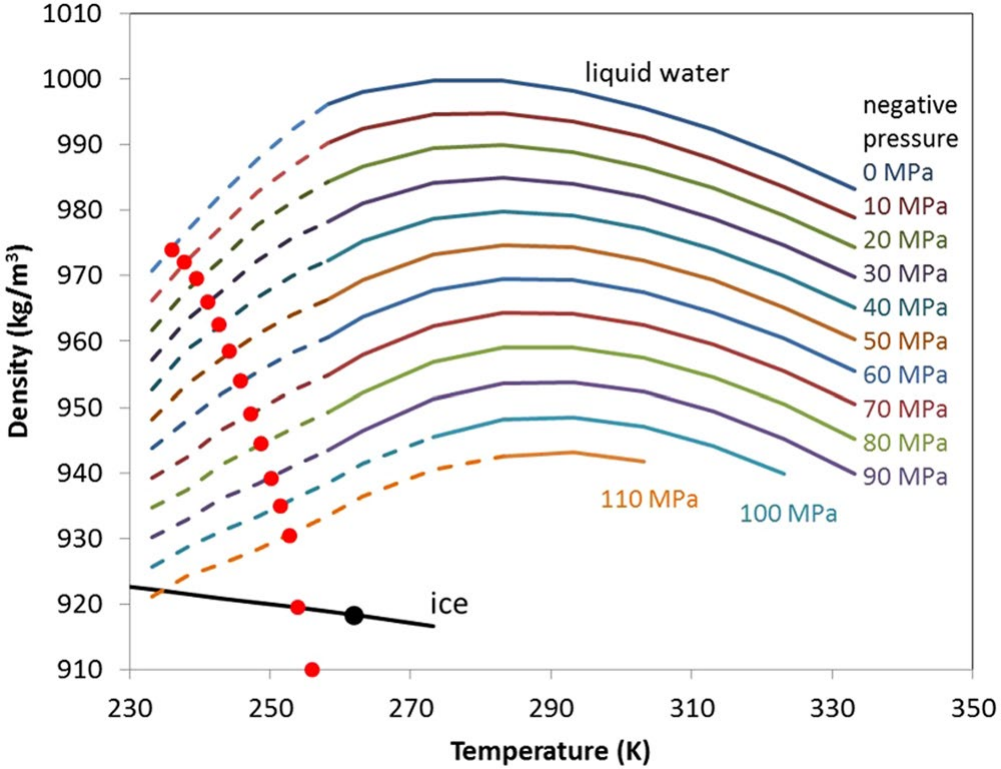
Density as a function of temperature for isobars from 0 to −110 MPa. The solid part of the isobars are the measurements by Pallares *et al*.^13^, the dashed portion an extrapolation. The black solid line gives the density of ice^52^. The red dots indicate for each isobar the homogeneous ice nucleation temperature for a nucleation rate coefficient of 10^8^ cm^−3^s^−1^ for the pressure of the isobar derived by shifting the melting curve by 307 MPa to lower pressure as explained in the theory section. The black dot on the ice density line indicates the temperature above which cavitation instead of freezing is expected to occur to relax the exerted negative pressure.

Recently, Pallares *et al*.^13^ determined experimentally an equation of state for water which confirms that the temperature of maximum density along isobars increases with decreasing pressure as shown in Fig. 2. In the investigated pressure range from ambient pressure to −110 MPa, the density of stretched water is still above the density of ice. The red dots on the extrapolated portion of the isobars indicate the predicted homogeneous ice nucleation temperature at the pressure of the isobar. Based on Fig. 2, water freezes without volume change when it is stretched at 254 K, indicated by the red dot on the ice density line; it expands when it is stretched below 254 K, and contracts upon freezing when it is stretched above 254 K. Above 262 K, negative pressure triggers cavitation rather than ice nucleation (see Fig. 1). Since freezing needs less or even no additional expansion when it is induced by stretching the water, the work to form ice is reduced rendering stretched water more susceptible to ice nucleation than water under ambient pressure. Moreover, molecular dynamics simulations showed that four-coordinated water molecules prevail in domains of low-density amorphous water and identified large patches of four-coordinated water as the precursors of ice embryos^14^. The fraction of four-coordinated water molecules is likely to increase when the density of water decreases because of the applied tension, boosting the formation of four-coordinated ice-like patches by random fluctuations.

The way walls and immersed particles promote ice nucleation is usually seen as the ability of their surfaces to reduce the energy needed to build up the interface when an ice embryo develops^2,15^. However, they might give rise to an additional effect. When liquids are exposed to pressure fluctuations, surfaces focus pressure waves into local spikes of large positive and negative pressures. Instances of negative pressure might trigger the formation of critical ice clusters that develop into ice crystals. Shpak *et al*.^16^ showed experimentally and by numerical simulations that high pressure amplitude acoustic waves generate superharmonics while traveling through a medium. When the wavelengths of these superharmonics are of the order of particles immersed in the medium, they generate focusing spots of large negative pressure at the surface of the particles, which are stronger the higher the density of the particle is compared with the density of the medium. Such focusing spots of negative pressure occurring at ice-nucleating surfaces might strongly enhance their intrinsic ice nucleation efficiency.

Thus, the effect of negative pressure acting on its own or together with ice-nucleating surfaces needs to be considered when ice nucleation occurs in the absence of a temperature decrease. Over a century ago, the Earl of Berkeley^17^ indeed mentioned negative pressure as a reason for ice nucleation. This speculation was taken up again by Wylie^18^ forty years later. He proposed that extremely negative pressures raise the melting point of ice thereby yielding ice embryos. There have been recurrent reports of freezing triggered by impact, shock or ultrasonic waves. Over a century ago Barnes^19^ noted that violent agitation raised the freezing point of pure distilled water samples by 2–3 K to 269–270 K. Dorsey^20^ observed that supercooled water did not freeze when it was poured from one end to the other in an ampule but crystallized at up to 2 K warmer temperature when it was violently shaken. In another set of experiments, he observed freezing of supercooled water when surfaces were dragged over each other within the water ampule. Young and Van Sicklen^21^ found that the mechanical shock of a metal hammer falling on an anvil submerged in water causes freezing. Goyer *et al*.^22^ were able to raise the freezing temperature of supercooled water contained in tubes by about 2 K to up to 270 K by transmitting shock waves. Finally, sonocrystallization uses ultrasonic waves to freeze water.

### Sonocrystallization

Although the ability of ultrasonic vibrations to trigger freezing has been known for a long time, the underlying mechanism is still under debate^23^. Since the pressure amplitudes of the ultrasonic fields used for sonocrystallization are too weak to nucleate ice directly, air or vapor bubbles cavitated by the ultrasonic waves are usually considered to be involved. Bubble collapse generates a high pressure pulse of the order of 5 GPa followed by tension pulses during the rebound of the cavity and due to the reflection of the pressure wave from free surfaces^24,25^. Hickling *et al*.^24^ conjectured that the high pressure pulse is able to nucleate high-pressure forms of ice opening up a pathway to normal ice I. However, the stability range of ice I and the high pressure forms of ice for T > 253 K do not overlap in the pressure domain rendering heterogeneous nucleation of ice I on high-pressure ice unlikely, even more, if one considers the different crystal lattice parameters of ice I and high-pressure ice^26–28^. On the other hand, the tension pulses arising from the bubble collapse should be intense enough to nucleate ice I directly^29^. Most studies investigated sonocrystallization in large water volumes at temperatures above 260 K and observed an increase of the freezing temperature by up to 10 K^30–33^. Figure 1 shows that above 262 K the rate of homogeneous cavitation under tension is higher than the rate of homogeneous ice nucleation, which is in agreement with the finding that cavitation precedes ice nucleation during sonocrystallization in this temperature range. The concurrent occurrence of ice nucleation and cavitation may indicate that the tension pulse arising from bubble collapse is intense and sharp enough to nucleate both, bubbles and ice, or that bubble and ice form on impurities that either enhance bubble or ice nucleation. At 255 K, Barrow *et al*.^34^ observed ice nucleation concurrent with cavitation of water put under tension in a Berthelot tube, which shows that at this temperature, negative pressure is able to nucleate ice without a bubble collapse being involved.

### Droplet freezing due to negative pressure

Ice nucleation triggered by negative pressure might explain rapid cloud glaciation observed in maritime and tropical clouds with cloud top temperatures of 258–268 K occurring in the absence of sufficient numbers of ice-nucleating particles active in this temperature range^35–37^. While secondary ice nucleation is often inferred as an explanation for such discrepancies^38^, ice nucleation triggered by negative pressure might also be relevant. Frequently, cloud glaciation is preceded by the emergence of millimeter-sized raindrops^35,36,38^. Drops exceeding one millimeter start to oscillate. The extent of deformation increases with increasing drop size^39,40^ leading to turbulent mixing within the drops. When strongly oscillating drops contain particles, transient negative pressure occurring at the surface of ice-nucleating particles might trigger ice nucleation directly. A bubble might form within the droplet on a hydrophobic particle and induce ice nucleation when it collapses, leading to ice fragments, which may initiate the cascade mechanism of cloud glaciation. When the oscillations grow large enough, the drop develops a thin neck in the middle and may break up typically into two parts. This mode of breakup is called dumbbell mode. Droplet collisions lead to coalescence or breakup. Filament, sheet, and disc, also called bag breakup are discriminated depending on the geometric shapes assumed by the drop pairs after their initial contact and before breakup^41,42^. The created fragments vary in size and number depending on the breakup mode and the initial droplet sizes. When large drops are deformed into a bag during breakup, the bag membrane becomes thinner as it is stretched until holes form, possibly giving rise to high enough tension to trigger the freezing of some fragments when temperature is low enough. Breakup of raindrops in the bag mode can occur in the wake of other drops and lead to ice fragments thus initiating secondary ice production e.g. by rime splintering^38^. Koenig^43^ observed freezing of some fragments during breakup of large drops in the bag mode at 258–267 K in laboratory experiments leading him to hypothesize that this mechanism may supply a link in the understanding of cloud glaciation. This conclusion was questioned by Edwards *et al*.^44^ because in their experiments millimeter-sized drops suspended in a cloud chamber at 263 K failed to produce ice fragments although the shock waves disintegrated them into many droplets. However, when a mechanical shock of similar intensity was exerted to drops sandwiched between films, the fragments froze even at 268 K. This highlights the enhancing effect of surfaces for dynamic ice nucleation.

## Conclusions and Outlook

Given the present state of knowledge, scenarios of ice nucleation induced by negative pressure are speculative but not altogether unconstrained. The relationship between negative pressure and ice nucleation temperature given in Fig. 1 provides a guidance to design freezing experiments applying negative pressure to trigger ice nucleation. Techniques to generate negative pressure in small volumes need to be developed and optimized to investigate the influence of negative pressure as a function of supercooling in controlled experiments. Simulations are needed to determine the peak negative pressures generated by mechanical shock, during the disruption of drops and due to bubble collapse. Molecular dynamics simulations may help to explore the effect of a decreased water density on melting and freezing of water and show how extended in time and space tension pulses need to be to trigger ice nucleation.

## Theory

### Ice nucleation

Classical nucleation theory views nucleation as an activated process that needs to overcome an energy barrier^45,46^. The free energy change for ice nucleation is given as

1 $${\rm{\Delta }}{G}_{iw}=4\pi {r}_{i}^{2}{\sigma }_{iw}-\frac{4\pi {r}_{i}^{3}}{3{\nu }_{i}}({\mu }_{w}-{\mu }_{i}).$$

The first term describes the work needed to build up the solid-liquid interface with an interfacial tension of *σ*_*iw*_ of the evolving ice crystal of size *r*_*i*_, the second term accounts for the energy released when water molecules become incorporated into the solid phase. Here, *ν*_*i*_ stands for the molecular volume of ice, *µ*_*i*_ and *µ*_*w*_ for the chemical potentials of ice and water, respectively. The difference in the chemical potentials of water molecules in ice and liquid water can be expressed as the ratio of vapor pressures above liquid water (*p*_*w*_) and ice (*p*_*i*_):

2 $${\mu }_{w}-{\mu }_{i}=kTln\frac{{p}_{w}}{{p}_{i}}$$

with *k* being the Boltzmann constant and *T* the absolute temperature. Ice clusters are thought to form randomly and dissipate unless thermal fluctuations become large enough to overcome the energy barrier *ΔG*_*iw,cr*_ and ice embryos of critical size *r*_*i,cr*_ form:

3 $${r}_{i,cr}=\frac{2{\nu }_{i}{\sigma }_{iw}}{kTln({p}_{w}/{p}_{i})}\,\,\,\,{\rm{a}}{\rm{n}}{\rm{d}}\,\,\,\,{\rm{\Delta }}{G}_{iw,cr}=\frac{16\pi }{3}\frac{{\nu }_{i}^{2}{\sigma }_{iw}^{3}}{{(kTln({p}_{w}/{p}_{i}))}^{2}}.$$

The nucleation rate coefficient (per volume and time) is formulated as an activated process:

4 $${j}_{iw,hom}=Z\frac{kT}{h}\exp (-\frac{{\rm{\Delta }}{F}_{diff}}{kT}){n}_{v}\exp (-\frac{{\rm{\Delta }}{G}_{iw,cr}}{kT}),$$

where *h* is the Planck constant, *ΔF*_*diff*_. is the activation energy for the transfer of a water molecule across the water-ice boundary, *n*_v_ is the number density of water molecules and *Z* is a correction factor^46^. The nucleation rate shows a steep increase with decreasing temperature. CNT parameterizations^46,47^ predict a 1 mm^3^ water volume to freeze within a second between 238 to 239 K, while a 10^9^ times smaller volume of 1 µm^3^ needs cooling to 234–235 K to freeze at the same rate. Critical nucleus sizes at these temperatures are calculated to be about 10 nm^3^.

In Fig. 1, the CNT parameterization by Zobrist *et al*.^47^ is used to calculate the temperature for which the nucleation rate coefficient equals 10^8^ cm^−3^s^−1^ in pure water (i.e. about 236 K). In a second step the offset in pressure is determined needed to shift the melting curve to lower pressure such that it passes through the temperature at which a nucleation rate coefficient of 10^8^ cm^−3^s^−1^ is realized for ambient pressure, resulting in ΔP = 307 MPa.

In case of heterogeneous ice nucleation, the energy barrier to form an ice embryo of critical size is reduced leading to the following expression for the ice nucleation rate coefficient

5 $${j}_{iw,het}=Z\frac{kT}{h}\exp (-\frac{{\rm{\Delta }}{F}_{diff}}{kT}){n}_{s}\,\exp (-\frac{{\rm{\Delta }}{G}_{iw,cr}{f}_{iw,het}}{kT}).$$

Here, the number density of water molecules *n*_*v*_ (~10^22^ cm^−3^) is replaced by the number density of water molecules at the nucleus/water interface *n*_*s*_ (~10^15^ cm^−2^) which reduces the pre-factor by 10^7^. The energy barrier to nucleation is reduced by a scaling factor *f*_*iw,het*_ describing the change of the Gibbs free energy as a function of the contact angle *α*_*is*_ of an ice embryo developing on an ice-inducing surface immersed in water:

6 $${f}_{iw,het}\,=\frac{1}{4}(2+\,\cos \,{\alpha }_{is})\,{(1-\cos {\alpha }_{is})}^{2}.\,$$

When *α*_*is*_ is low, the surface has a high preference for ice compared with water. For *α*_*is*_ = 0, the energy barrier is even reduced to zero.

### Bubble nucleation

The homogeneous nucleation of a bubble can be described by CNT using similar expressions as for the nucleation of a solid^45^. The evolution of the free energy during bubble formation under negative pressure is given by

7 $${\rm{\Delta }}{G}_{bw}=4\pi {r}_{b}^{2}{\sigma }_{bw}-\frac{4\pi {r}_{b}^{3}}{3{\nu }_{l}}({\mu }_{l}-{\mu }_{b}).$$

Here, the index *b* refers to the bubble. Thus, *r*_*b*_ is the radius of the evolving bubble, *µ*_*b*_ is the chemical potential of water molecules in the bubble, while *µ*_*l*_ is the chemical potential of the molecules in liquid water at normal pressure and *ν*_*l*_ is the molecular volume of liquid water. Again, the difference in chemical potential can be expressed as the ratio of vapor pressures of liquid water, *p*_*l*_, and vapor pressure in the bubble, *p*_*b*_:

8 $${\mu }_{l}-{\mu }_{b}=kTln\frac{{p}_{l}}{{p}_{b}}$$

The change of vapor pressure in dependence of the pressure applied to the liquid, *P*_*ex*_, is described by the Laplace-Kelvin equation:

9 $$kTln\frac{{p}_{l}}{{p}_{b}}={\nu }_{l}({p}_{l}-{P}_{ex})$$

thus the critical radius and the energy barrier are given as:

10 $${r}_{b,cr}=\frac{2{\sigma }_{bw}}{({p}_{l}-{P}_{ex})}\,\,\,\,{\rm{a}}{\rm{n}}{\rm{d}}\,\,\,\,{\rm{\Delta }}{G}_{bw,cr}=\frac{16\pi {\sigma }_{bw}^{3}}{3{({p}_{l}-{P}_{ex})}^{2}}$$

the expression for the rate of homogeneous bubble nucleation per volume and time is^45^

11 $${j}_{bw,hom}={n}_{v}{(\frac{2{\sigma }_{bw}}{\pi mB})}^{1/2}\exp (\frac{-\Delta {G}_{bw,cr}}{kT})$$

here, *m* is the mass of a molecule and *B* is a coefficient with a value of 1 for chemical equilibrium and 2/3 for mechanical equilibrium. CNT is the simplest approach to calculate the cavitation pressure. More sophisticated calculations based on molecular dynamics simulations and density functional theory predictions give values of the same order of magnitude but some of them with different temperature dependences^9^. Figure 1 shows a parameterization of classical nucleation theory for cavitation using Eq. (11). To calculate the prefactor, the temperature dependent parameterization of the surface tension given by the IAPWS (International Association for the Properties of Water and Steam) correlation^48,49^ was used, which was multiplied with the correction factor *k*_*σ*_ following Shneider *et al*.^50^:

12 $${\sigma }_{bw}(T)=B{\tau }^{\mu }(1+b\tau ){k}_{\sigma }$$

with *τ* = 1 − *T/T*_*c*_ being the dimensionless distance from the critical temperature *T*_*c*_ = 647.096 K, *µ* = 1.256 being a universal critical exponent, and coefficients *B* and *b* having values of 235.8 mNm^−1^ and −0.625, respectively. The correction factor *k*_*σ*_ is introduced to describe the radius dependence of the surface tension^50^:

13 $${k}_{\sigma }=\frac{1}{1+2\delta /{R}_{cr}}$$

where *δ* = −0.047 nm is the Tolman coefficient^12^ and *R*_*cr*_ = 2*σ*_*bw*_(*p*_*l*_ *−P*_*ex*_). The coefficient *B* was set to 1 and the number density of water molecules in the liquid phase was taken as *n*_*v*_ = 3.2944·10^28^ m^−3^.

As for ice nucleation, cavitation usually occurs on surfaces that reduce the energy needed to build up the surface of the evolving bubble. CNT describes the heterogeneous bubble nucleation at a rigid interface^45^ as

14 $${j}_{bw,het}={n}_{s}(\frac{1-m}{2}){(\frac{2{\sigma }_{bw}}{\pi mB{f}_{bw,het}})}^{1/2}\exp (\frac{-{\rm{\Delta }}{G}_{bw,cr}{f}_{bw,het}}{kT}).$$

Here the scaling factor for bubble nucleation is a function of the contact angle between water and the surface *α*_*ws*_

15 $${f}_{bw,het}\,=\frac{1}{4}(2+\,\cos (\pi -{\alpha }_{ws})){(1-\cos (\pi -{\alpha }_{ws}))}^{2}\,.\,$$

The prefactor for heterogeneous nucleation is smaller than the one for homogeneous nucleation by a factor of 10^7^. Hydrophobic surfaces are expected to promote cavitation while it is still not well known what surface properties are needed to induce ice nucleation. Surface irregularities, such as grain boundaries, ledges, cracks and scratches are often considered as preferred locations for nucleation of bubbles and ice^45^.

## References

[1] Nagy ZK, Braatz RD (2012). Advances and new directions in crystallization control. Annu. Rev. Chem. Biomol. Eng..

[2] Vali G, DeMott PJ, Möhler O, Whale TF (2015). Technical note: a proposal for ice nucleation terminology. Atmos. Chem. Phys..

[3] Mossop SC (1955). The freezing of supercooled water. P. Phys. Soc. B.

[4] Koop T, Luo BP, Tsias A, Peter T (2000). Water activity as the determinant for homogeneous ice nucleation in aqueous solutions. Nature.

[5] Zobrist B, Marcolli C, Koop T, Peter T (2008). Heterogeneous ice nucleation in aqueous solutions: the role of water activity. J. Phys. Chem. A.

[6] Roedder E (1967). Metastable superheated ice in liquid-water inclusions under high negative pressure. Science.

[7] Briggs LJ (1950). Limiting negative pressure of water. J. Appl. Phys..

[8] Galloway WJ (1954). An experimental study of acoustically induced cavitation in liquids. J. Acous. Soc. Am..

[9] Caupin, F. & Stroock, A. D. The stability limit and other open questions on water at negative pressure. *Liquid Polymorphism, Book Series: Advances in Chemical Physics* 152, 51–80 (2013).

[10] Zheng Q, Durben DJ, Wolf GH, Angell CA (1991). Liquids at large negative pressures: water at the homogeneous nucleation limit. Science.

[11] Shmulovich KI, Mercury L, Thiéry R, Ramboz C, El Mekki M (2009). Experimental superheating of water and aqueous solutions. Geochim. Cosmochim. Acta.

[12] Azouzi MEM, Ramboz C, Lenain J-F, Caupin F (2013). A coherent picture of water at extreme negative pressure. Nat. Phys..

[13] Pallares, G., Gonzalez, M. A., Abascal, J. L. F., Valeriani, C. & Caupin, F. Equation of state for water and its line of density maxima down to −120 MPa. *Phys. Chem. Chem. Phys.* **18**, 5896−5900 (2016). 10.1039/c5cp07580g 26840756

[14] Moore EB, Molinero V (2011). Structural transformation in supercooled water controls the crystallization rate of ice. Nature.

[15] Kaufmann L, Marcolli C, Luo B, Peter T (2017). Refreeze experiments of water droplets containing different types of ice nuclei interpreted by classical nucleation theory. Atmos. Chem. Phys..

[16] Shpak O (2014). Acoustic droplet vaporization is initiated by superharmonic focusing. Proc. Natl. Acad. Sci. USA.

[17] Berkeley (1912). Earl of. Solubility and supersolubility from the osmotic standpoint. Phil. Mag..

[18] Wylie RG (1953). The freezing of supercooled water in glass. Proc. Phys. Soc. London.

[19] Barnes, H. T. Ice Formation. J. *Wiley*, p 96 (1906).

[20] Dorsey NE (1948). The freezing of supercooled water. Trans. Am. Phil. Soc., New Series.

[21] Young SW, Van Sicklen WJ (1913). The mechanical stimulus to crystallization. Amer. Chem. Soc. J..

[22] Goyer GG, Bhadra TC, Gitlin S (1965). Shock induced freezing of supercooled water. J. Appl. Meteor..

[23] Sander JRG, Zeiger BW, Suslick KS (2014). Sonocrystallization and sonofragmentation. Ultrasonics Sonochemistry.

[24] Hickling R (1965). Nucleation of freezing by cavity collapse and its relation to cavitation damage. Nature.

[25] Williams PR, Williams PM, Brown SWJ (1997). Pressure waves arising from the oscillation of cavitation bubbles under dynamic stressing. J. Phys.. D: Appl. Phys..

[26] Kanno H, Speedy R, Angell CA (1975). Supercooling of water to −92 °C under pressure. Science.

[27] Mishima O (1996). Relationship between melting and amorphization of ice. Nature.

[28] Salzmann CG, Radaelli PG, Slater B, Finney JL (2011). The polymorphism of ice: five unresolved questions. Phys. Chem. Chem. Phys..

[29] Hunt JD, Jackson KA (1966). Nucleation of solid in an undercooled liquid by cavitation. Appl. Phys..

[30] Inada T, Zhang X, Yabe A, Kozava Y (2001). Active control of phase change from supercooled water to ice by ultrasonic vibration 1. Control of freezing temperature. Int. J. Heat Mass Transfer.

[31] Hozumi T, Saito A, Okawa S, Matsui T (2002). Freezing phenomena of supercooled water under impacts of ultrasonic waves. Int. J. Refrigeration.

[32] Zhang X, Inada T, Tezuka A (2003). Ultrasonic-induced nucleation of ice in water containing air bubbles. Ultrasonics Sonochem..

[33] Chow R, Blindt R, Chivers R, Povey M (2005). A study on the primary and secondary nucleation of ice by power ultrasound. Ultrasonics.

[34] Barrow MS, Williams PR, Chan H-H, Dore JC, Bellissent-Funel M-C (2012). Studies of cavitation and ice nucleation in ‘doubly-metastable’ water: time-lapse photography and neutron diffraction. Phys. Chem. Chem. Phys..

[35] Lawson RP, Woods S, Morrison H (2015). The microphysics of ice and precipitation development in tropical cumulus clouds. J. Atmos. Sci..

[36] Koenig LR (1963). The glaciating behavior of small cumulonimbus clouds. J. Atmos. Sci..

[37] Petters MD, Wright TP (2015). Revisiting ice nucleation from precipitation samples. Geophys. Res. Lett..

[38] Field PR (2017). Secondary ice production: current state of the science and recommendation for the future. Meteor. Monograph, Amer. Meteor. Soc..

[39] Szakáll M, Mitra SK, Diehl K, Borrmann S (2010). Shapes and oscillations of falling raindrops – a review. Atmos. Res..

[40] Beard KV, Bringi VN, Thurai M (2010). A new understanding of raindrop shape. Atmos. Res..

[41] Low TB, List R (1982). Collision, Coalescence and breakup of raindrops. 2. Parameterization of fragment size distributions. J. Atmos. Sci..

[42] Emersic C, Connolly PJ (2011). The breakup of levitating water drops observed with a high speed camera. Atmos. Chem. Phys..

[43] Koenig LR (1965). Drop freezing through drop breakup. J. Atmos. Sci..

[44] Edwards GR, Evans LF, Hamann SD (1969). Nucleation of ice by mechanical shock. Nature.

[45] Blander M, Katz JL (1975). Bubble nucleation in liquids. AIChe J..

[46] Ickes L, Welti A, Hoose C, Lohmann U (2015). Classical nucleation theory of homogeneous freezing of water: thermodynamic and kinetic parameters. Phys. Chem. Chem. Phys..

[47] Zobrist B, Koop T, Luo BP, Marcolli C, Peter T (2007). Heterogeneous ice nucleation rate coefficient of water droplets coated by a nonadecanol monolayer. J. Phys. Chem. C.

[48] Hruby J, Vinš V, Mares R, Hykl J, Kalova J (2014). Surface tension of supercooled water: no inflection point down to −25 °C. J. Phys. Chem. Lett..

[49] Vinš V, Fransen M, Hykl J, Hruby J (2015). Surface tension of supercooled water determined by using a counterpressure capillary rise method. J. Phys. Chem. B.

[50] Shneider MN, Pekker M (2013). Cavitation in dielectric fluid in inhomogeneous pulsed electric field. J. Appl. Phys..

[51] Henderson SJ, Speedy RJ (1987). Melting temperature of ice at positive and negative pressures. J. Phys. Chem..

[52] Haynes, W. M. Ed.: CRC Handbook of Chemistry and Physics, 96th ed.; CRC Press: Boca Raton (2015).

